# Potential common targets of music therapy intervention in neuropsychiatric disorders: the prefrontal cortex-hippocampus -amygdala circuit (a review)

**DOI:** 10.3389/fnhum.2025.1471433

**Published:** 2025-02-03

**Authors:** Yuqin Dan, Ying Xiong, Danghan Xu, Yuqi Wang, Meng Yin, Pengwei Sun, Yi Ding, Ziyun Feng, Peng Sun, Weili Xia, Gongchang Yu, Li Li

**Affiliations:** ^1^The College of Rehabilitation Medicine, Shandong University of Traditional Chinese Medicine, Jinan, China; ^2^School of Traditional Chinese Medicine, Shandong University of Traditional Chinese Medicine, Jinan, China; ^3^Rehabilitation Center, The First Affiliated Hospital of Guangzhou University of Chinese Medicine, Guangzhou, China; ^4^Shandong First Medical University & Shandong Academy of Medical Sciences, Shandong Academy of Occupational Health and Occupational Medicine, Jinan, China; ^5^School of Pharmacy, Shandong University of Traditional Chinese Medicine, Jinan, China; ^6^Department Rehabilitation Medicine, The Second Affiliated Hospital of Shandong University of Traditional Chinese Medicine, Jinan, China; ^7^Innovation Research Institute of Chinese Medicine, Shandong University of Traditional Chinese Medicine, Jinan, China; ^8^Shandong Mental Health Center, Shandong University, Jinan, China; ^9^Neck-Shoulder and Lumbocrural Pain Hospital of Shandong First Medical University, Shandong First Medical University & Shandong Academy of Medical Sciences, Jinan, China

**Keywords:** neuropsychiatric disorders, music therapy, the prefrontal cortex-hippocampus -amygdala circuit, potential mechanisms, common targets

## Abstract

As life becomes more stressful, neurological disorders, psychiatric disorders, and comorbidities of the two are becoming more and more of a concern. Multiple neuropsychiatric disorders share the same mental and somatic dysfunction and may involve common brain circuits and mechanistic targets. Music therapy, as an art form with proven efficacy, low cost and few side effects, is promoted for use in interventions for neuropsychiatric disorders. This may be closely related to the release of signaling molecules such as monoamine neurotransmitters, the glutamatergic system, the gut-microbiota-brain axis, pro-inflammatory cytokines and the endogenous opioid peptide system. However, fewer studies have mentioned the main targets of music to promote functional changes in brain regions. Therefore, this paper is a review of the mechanisms by which music therapy interacts with the prefrontal cortex-hippocampus-amygdala circuit through the aforementioned molecules. It is also hypothesized that glial cells, mitochondria and microRNAs are microscopic targets for musical intervention in neuropsychiatric disorders. The aim is to give new ideas for future research into the biological mechanisms of music therapy intervention in neuropsychiatric disorders.

## 1 Introduction

The World Health Organization has documented that neurological and psychiatric disorders rank among the three leading causes of mortality worldwide, with a significant impact on disability, particularly in the European region where they account for an alarming 45.9% of disability rates (GBD 2021 Causes of Death Collaborators, 2024). Concurrently, mental health disorders often co-occur with a range of neurological disorders, including epilepsy, migraines, Alzheimer’s disease, Parkinson’s disease, essential tremor, and cerebrovascular accidents(stroke) (McGrath et al., 2023). These neurological and psychiatric disorders that co-occur are collectively referred to as Neuropsychiatric Disorders (NPDs), which are chronic conditions prevalent worldwide (Levy and Pasca, 2023).

NPDs typically arise from the complex interplay between external stressors and genetic factors, leading to progressive alterations in brain physiology and the development of functional disabilities (Gupta et al., 2023; Li et al., 2022). Statistics reveal a high comorbidity rate between the neurological disease and mental disorder under consideration, with the risk of developing a mental disorder increasing alongside the duration of the neurological condition (Maxwell et al., 2022). For instance, depression may emerge in 53% of patients within three months following a stroke, and sleep disturbances, hallucinations, and delusions are prevalent in 71.0% of individuals with Alzheimer’s disease (Eikelboom et al., 2022; Liu et al., 2023). Furthermore, between 20 and 30% of Parkinson’s patients experience depression, anxiety, or cognitive decline (Cong et al., 2022). The combined effect of these conditions not only exacerbates the patients’ loss of autonomy but also intensifies the financial strain on households and may even contribute to a reduced life expectancy (Neue et al., 2022). This underscores an urgent need for a broader societal awareness and response to these comorbidities.

The etiology of NPDs is relatively complex, involving the interplay of genetic, neurobiological, and environmental factors (Shahcheraghi et al., 2023). Current research indicates that monoamine neurotransmitters, pro-inflammatory cytokines, amino acid neurotransmitters, chemokine receptors, the endogenous opioid peptide system, and the gut microbiome-brain axis are all potential mechanisms underlying NPDs (Conti and Shaik-Dasthagirisaheb, 2015; Hassamal, 2023; Malan-Muller et al., 2022; Saggu et al., 2024; Torres-Berrio and Nava-Mesa, 2019). Moreover, these research mechanisms all seem to converge on the “prefrontal cortex(PFC)-hippocampus(Hip)-amygdala(Amy)” circuit, highlighting its potential role in the development and manifestation of these conditions (Krishnamoorthy, 2007). For instance, Song J et al. discovered that the connections between the amygdala and other brain regions, such as the Hip, as well as the activity within the amygdala, lead to various neuropathological and neuropsychiatric issues (Song, 2023). Maynard KR and colleagues identified common genes across various NPDs in the dorsolateral PFC through the analysis of post-mortem brain tissue, suggesting that abnormal gene expression in this region could be a significant factor in the development of NPDs such as schizophrenia and autism (Maynard et al., 2021). Extending this line of research, similar conclusions have been drawn from gene expression studies in the amygdala, highlighting the potential role of gene expression abnormalities in the amygdala in the pathogenesis of these disorders (Ou et al., 2023). Additionally, a previous review study has posited that the dynorphin/κ-opioid receptor system, which is associated with the amygdala circuitry, exerts a favorable regulatory effect on NPDs (Limoges et al., 2022). Thus, the structure and functional status of the “PFC-Hip-Amy” circuit may play a common and crucial regulatory role in the amelioration of various NPDs. Specific research indicates that the PFC plays an essential role in motor control and emotional regulation (Chafee and Heilbronner, 2022). Changes in the volume of the Hip are considered potential biomarkers related to memory function (Ott et al., 2019). Meanwhile, the Amy is typically involved in stress-induced NPDs (Mazzitelli et al., 2024).

At present, most targeted drug therapies for neurological and psychiatric disorders in clinical practice are based on the specific molecular mechanisms mentioned above. However, these treatments often come with significant side effects that can greatly impact patients’ wellbeing (Foster et al., 2021). Adverse reactions such as hepatic and renal impairment, cardiac arrhythmias, and malignant syndromes have been documented in patients undergoing drug therapy (Anand et al., 2020). Additionally, there is considerable individual variability in response and a lack of robust evidence for the efficacy of some medications (Ilomaki et al., 2020). This has led to a growing need for non-pharmacological therapies. Among these, music therapy (MT) has emerged as a significant complementary treatment for NPDs (Sikkes et al., 2021). MT has been shown to markedly improve anxiety and depression in patients with neurological or psychiatric conditions, as well as to enhance cognitive function and daily living skills (Lin et al., 2023). The benefits of MT, including fewer side effects, lower costs, and greater safety, have increasingly captured the interest of clinical practitioners (Yin et al., 2022). Since the 21st century, research into the effects of music on brain function has gained significant traction, with a particular focus on the interaction between music and the “PFC-Hip-Amy” circuit (Skouras et al., 2014). Studies suggest that music training can strengthen the structural and functional connectivity within the “PFC-Hip-Amy” circuitry, leading to improved cognitive abilities and emotional regulation (Koelsch and Skouras, 2014; Pant et al., 2022). Moreover, music has been shown to boost the efficiency of brain networks, allowing for more effective information processing (Speranza et al., 2022). These insights not only enrich our comprehension of the nexus between music and brain function but also lay a scientific foundation for the therapeutic use of music in addressing NPDs (Gassner et al., 2022).

Therefore, this paper will take the perspective of disease dysfunction. Summarize the clinical efficacy of MT for NPDs and attempt to explore the key brain circuits (PFC-Hip-Amy), mechanisms, and potential targets involved.

## 2 Research progress of music therapy intervention in neuropsychiatric disorders

Previous literature reviews have extensively covered the application of music therapy in neurological and psychiatric disorders, focusing primarily on its impact on conditions such as stroke, dementia, Parkinson’s disease, as well as depression and anxiety (Aalbers et al., 2017; Matziorinis and Koelsch, 2022; Sihvonen et al., 2017). However, there remains a significant gap in the synthesis of research regarding the comorbidity of NPDs. This review will integrate the latest research findings to provide a comprehensive analysis of the therapeutic effects of music therapy in the treatment of NPDs.

### 2.1 Definition and overview of music therapy

**FIGURE 1:**
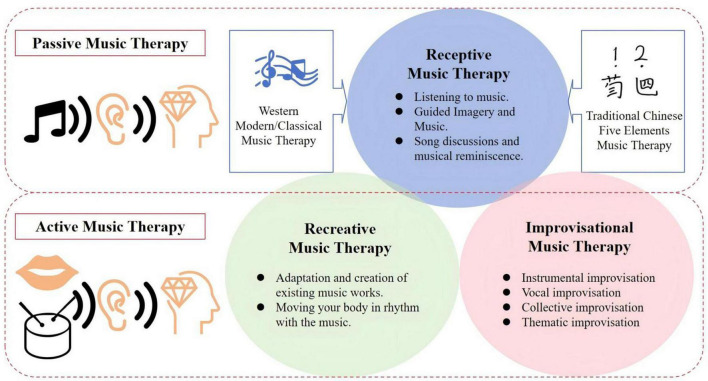
Classifications of music therapy. MT is categorized into three main types. (1) Receptive music therapy, which involves directed listening. (2) Recreative music therapy, where patients actively adapt or create music. (3) Improvisational music therapy, focusing on spontaneous instrumental performance based on inner feelings. These classifications reflect the diverse ways MT can be applied to improve mood, cognition, and behavior in various patient populations.

Music therapy (MT) is defined as an artistic healing process that activates or inhibits specific areas of the brain through active or passive forms of musical activity, including listening to music, songwriting, listening, singing, playing, and rhythmic stimulation, in order to improve an individual’s mood, cognition, and behavior (Bhandarkar et al., 2024). The therapy was formally established as an applied discipline at Michigan State University in the United States in 1944, and has since been widely adopted in developed countries due to its remarkable efficacy in the treatment of psychosomatic diseases (Gao et al., 2019). Through the continuous development, MT is divided into receptive music therapy (such as directed listening to music), recreative music therapy (which involves actively participating in the adaptation and creation of existing musical works), and improvisational music therapy (referring to the patient’s spontaneous instrumental performance based on their inner feelings) (Lam et al., 2020). It is noteworthy that a systematic evaluation has demonstrated that Recreative Music Therapy is more effective in terms of desensitization, analgesia, stress reduction, and hypnosis compared to the other two types of music therapy in treating dementia patients with disabling disuse (Tsoi et al., 2018). Additionally, while therapists can select from a broad range of performance instruments, forms, and vocal works for musical themes, they typically take into account the patient’s ethnicity, cultural environment, and educational background to boost the patient’s engagement and compliance in the therapy process (Wilson et al., 2016). Conclusively, MT stands out for its affordability, proven safety, and extensive applicability across a wide range of diseases and age groups, underscoring its value for broader implementation in community, home, and hospital settings. It consistently demonstrates significant benefits and untapped potential in the effective treatment of various NPDs, making it a compelling option for enhancing patient care and rehabilitation (Figure 1).

### 2.2 Intervention effect of music therapy in neuropsychiatric disorders

Distinct from pharmacological treatments, MT does not directly target the diseases themselves. Instead, it enhances the psychological and physical functioning of patients with NPDs, thereby improving their symptoms and overall quality of life (Raglio, 2017). This enhancement encompasses advancements in cognitive, psychosocial, behavioral, and motor functioning (Brancatisano et al., 2020). Scholars have observed that the efficacy of MT can surpass that of conventional talk therapy in certain instances (Beck et al., 2021). Consequently, the following discussion will explore the efficacy of MT intervention in NPDs from the vantage point of its impact on both physical and mental functioning.

#### 2.2.1 Music therapy improves cognitive and behavioral skills

Cognitive impairment stands out as a prevalent functional disability in NPDs, stemming from neurological damage or degenerative conditions like stroke, Alzheimer’s disease, Parkinson’s disease, and brucellosis (Dotson and McClintock, 2020; Nalini et al., 2012). Such impairments frequently result in unfavorable prognoses for patients and can precipitate mental health issues, including delirium, depression, and anxiety (Sun et al., 2022). A study found that early intervention in MT has been found to slow down the disease process in patients with dementia, with significant efficacy in terms of overall cognitive status, attention, immediate or long-term memory, executive function, as well as gait speed, gait and stride length (Dominguez-Chavez et al., 2019). At the same time, caregivers of dementia patients experienced a reduction in their fatigue and a subsequent increase in their productivity as a result of music therapy (Holden et al., 2019). In addition, the therapist can maximize the efficacy of MT by controlling the duration of treatment, environmental conditions or the form of intervention. For example, Sharma A and other scholars gave 30–40 min of music to AD patients early in the morning for 4 consecutive weeks, which could accompany circadian rhythms and thus increase serum melatonin levels, thus improving sleep quality and cognitive performance of the patients (Sharma et al., 2021). Another study showed that vocal music plays a positive role in the rehabilitation of stroke patients by driving the temporoparietal network to actively process emotional responses through the dual superimposition of sound input and speech output, which in turn facilitates the remodeling of the structure and function of the language center (Sihvonen et al., 2020). Thus, recreational MT promotes more expressive language skills and text comprehension, and is suitable for use in post-stroke aphasia and prosopagnosia, as well as in patients with dementia. However, the intervention period of MT has not been clarified in related studies, and the short-term intervention effect may present less neurological promotion and functional reconstruction in patients with more severe conditions.

On the other hand, MT has demonstrated significant advantages in improving abnormal behaviors in patients with NPDs. A study focusing on autism spectrum disorder has demonstrated that MT not only effectively reduces aggressive behaviors in children and adolescents but also bolsters their self-control capabilities (Ye et al., 2021). Additionally, MT can indirectly foster social participation in children with autism by enhancing their reading, speech, and writing skills, thereby equipping them with the potential for independent living (Sharda et al., 2019). In the realm of dementia care, personalized ethnic and cultural MT has been found to decrease the frequency of manic and aggressive episodes in Alzheimer’s disease patients (McCreedy et al., 2021). In the treatment of aphasia, rhythmic melodic intonation therapy has been instrumental in improving verbal functions and communication skills in post-stroke patients with non-fluent aphasia, as well as in alleviating their anxiety and depression (Liu Q. et al., 2022). Furthermore, for dysphagia resulting from neurological disorders, a combination of breathing exercises, vocalization, and singing has been shown to be beneficial (Kim et al., 2023). It has been proposed that MT may even surpass pharmacotherapy in managing behavioral disorders (Watt et al., 2019). This finding, although not universally confirmed, is sufficient to suggest that MT has great potential and has demonstrated therapeutic value in a range of NPDs.

In summary, MT has demonstrated the capacity to address a broad spectrum of conditions. Beyond the disorders previously discussed, music has been shown to be particularly effective in treating anorexia nervosa, bulimia, gait abnormalities, and obsessive-compulsive behavior (Chang et al., 2023; Park et al., 2019; Truong et al., 2021). The influence of MT on human behavior is typically mediated through the “emotion-cognition-behavior” chain, suggesting a gradual and indirect impact. However, the question of whether music can directly alter human behavior remains open for investigation. It is also unclear if the efficacy of MT across different diseases is mediated by the same neural pathways. The potential for MT to serve as a substitute for cognitive and behavioral therapies in psychotherapy is another area that warrants further exploration. These queries necessitate ongoing research to elucidate the precise mechanisms and applications of MT.

#### 2.2.2 Music therapy alleviate mood disorders

Amidst escalating societal pressures, there is a noted rise in the prevalence of primary depression and anxiety disorders, along with the potential for neurodegenerative diseases to trigger depressive states and despair among patients. In such contexts, Music Therapy (MT) has emerged as a therapeutic modality with distinct benefits (Li et al., 2020). Extensive research has consistently demonstrated that both individual and group Music Therapy (GMT) can provide immersive enjoyment and energetic motivation, enhancing social interaction while alleviating loneliness and disorientation (Aalbers et al., 2017). Music relaxes the mind and body in a short period of time, obtaining a long-lasting effect (Tang et al., 2020). Erkkilä et al. uses a combination of music therapy and slow-paced breathing modification therapy as a way to enhance the effectiveness of treatment when improving depression in office workers (Erkkila et al., 2021). In addition, MT can also improve anxiety and depression during the rehabilitation treatment of postpartum, cancer patients, postoperative patients, and other groups to a certain extent, and accelerate the speed of recovery of patients (Chirico et al., 2020; Daykin et al., 2018; Yang W. J. et al., 2019).

Music has been recognized for its dual capacity to elevate the spirits of those experiencing depression and to temper excessive emotional responses, thus exerting a bidirectional regulatory effect on emotions. Research indicates that the involvement of MT has led to an improvement in dissociative identity disorder in patients with bipolar disorder, which effectively reduces the incidence of mania and provides lasting efficacy (Greenway et al., 2021). Other studies have shown that group music therapy (GMT) in combination with standard pharmacological treatment is effective in reducing or controlling the dose of antipsychotics in patients with mania and bipolar disorder (Degli Stefani and Biasutti, 2016). Interestingly, cultural connotation of music are also crucial in treatment efficacy. For example, under the influence of Eastern culture, Chinese patients tend to experience better therapeutic outcomes when listening to traditional Chinese music based on the Five Elements theory compared to Western music (Liao et al., 2023). Therefore, when patients listen to music that is indigenous to their culture, they may experience a stronger emotional bond and sense of belonging. This emotional engagement can stimulate a variety of psychological and physiological responses that contribute to overall health and recovery. Essentially, the efficacy of music therapy for Chinese patients depends not only on the music itself but also on how it interacts with the listener’s cultural background and psychological state. This underscores the importance of considering cultural factors in therapeutic practices to maximize their effectiveness.

#### 2.2.3 Music therapy improves motor control ability

Beyond its cognitive, emotional, and spiritual benefits, music also exerts a positive influence on the motor system through the facilitation of neurofeedback (Thaut et al., 2014). MT enhances individuals’ motor control capabilities, particularly in enhancing gait performance and mitigating the risk of falls (Devlin et al., 2019). Within this context, the rhythmic component of music therapy is especially significant, playing a crucial role in the overall therapeutic process. Research has shown that the sensory input of the patient is facilitated, while the mobility of the patient is indirectly improved. This phenomenon occurs in relation to changes in overall brain function induced in the auditory cortex (Emmery et al., 2023). Moreover, the music rhythm prompted the enhancement of frontal-center-parietal-temporal lobe functional connectivity, which stimulated the cerebellum’s regulatory function to a certain extent, increased the innervated muscle strength, and the efficiency and intensity of the patient’s gait training were increased, which led to the improvement of gait performance (Calabro et al., 2019). Compared to passive listening to music, active playing of music enhances the ability to act with anticipatory awareness and better strengthens coordinated movement and behavioral control. For example, active playing of musical instruments by stroke patients can promote auditory-motor integration, coalesce emotional-motivational effects, relieve spasticity of the affected upper limb, and enhance fine motor skills of the fingers (Patania et al., 2020). Listening to group drumming in the morning can activate the corresponding motor nerves in PD patients to reduce their resting tremor (Smolensky et al., 2016). In addition, active music therapy in the form of drumming improves muscle strength, balance, proprioception and emotional state in children with corpus callosum hypoplasia, with positive efficacy of treating the body and mind together (Spak and Card, 2020).

**FIGURE 2:**
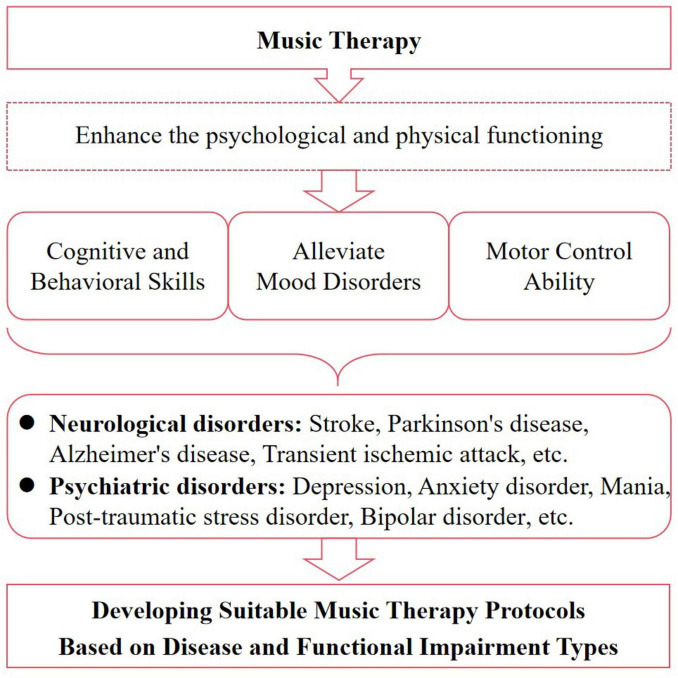
Music heals diseases by improving functionality. MT improves the quality of life for patients with NPDs by enhancing their psychological and physical functioning, including cognitive, psychosocial, behavioral, and motor aspects.

In conclusion, MT has demonstrated significant regulatory effects on various aspects of patients with NPDs, including cognitive ability, emotional state, behavioral control, and motor coordination. Furthermore, receptive music therapy is commonly used to treat psychiatric disorders, while re-creation music therapy and improvisation music therapy are particularly beneficial for cognitive and motor dysfunction in neurological disorders. Therefore, depending on the type of disease and dysfunction, a suitable music therapy program should be developed to intervene accordingly (Figure 2). However, despite the efficacy of MT in improving NPDs being confirmed in numerous clinical trials, the molecular mechanisms are not yet fully elucidated, and there is a lack of evidence regarding pathways or targets. This could be a hot area for future research.

## 3 Relationship between the “PFC-Hip-Amy”circuit and nueropsychiatric disorders

Non-invasive imaging has shown that the PFC, hippocampus and amygdala are interconnected and act in a coordinated manner through direct and indirect neural activity (Nawa and Ando, 2019). A large body of evidence suggests that the direct or indirect neural activity of this circuit regulates a wide range of social, cognitive, and affective functions (Chao et al., 2022). And dominates the process of “onset, progression, and recovery” of many neurological diseases and psychiatric disorders (Shin et al., 2022). The hippocampus and PFC are responsible for encoding the formation of conditioned memories and dominate the reproduction of memories after they have faded, affecting the patient’s functions such as remembering objects, recognizing orientation, and planning motor routes (Jobson et al., 2021; Park et al., 2021). In addition, coordinated oscillatory activity between the hippocampus and the amygdala has been linked to the formation, consolidation, and extinction of situational and emotional memories (Wang D. et al., 2023). The interaction between basal and dense fission in the posterior pallid amygdala can modulate motor behavior (Ghazizadeh et al., 2018). Therefore, abnormal functioning of this circuit disrupts cognitive-psychological and motor-behavioral functioning and is an important cause of many NPDs.

### 3.1 The “PFC-Hip-Amy” circuit influences the development of neurological diseases

In many neurodegenerative or injurious lesions, patients often present with decreased attention, spatial disorientation, memory loss, or abnormal postural control, which are significantly associated with abnormalities in the functional connectivity between the “PFC-Hip-Amy” circuits (Huang et al., 2016).

Specifically, damage to the PFC in stroke patients leads to the development of somatomotor dysfunction, whereas the degree of damage to astrocytes in the hippocampus is positively correlated with the degree of cognitive impairment in patients (Stancu et al., 2022). Therefore, it has been suggested that the further development of cognitive deficits after an ischaemic stroke can be prevented by reducing the leakage of G immunoglobulin in the hippocampus (Zhang et al., 2019). Others have suggested that increased levels of IL-1β, IL-6, and TNF-α in the hippocampus and amygdala, as well as amplification of GAT-1 and GAT-3, are the main causes of non-convulsive seizures after cerebral ischaemia (Banwinkler et al., 2022). In addition, atrophy of the PFC and hippocampus is commonly seen in Parkinson’s patients. Moreover, as the disease progresses, damage to neurons in the paracortical nucleus of the amygdala, the basal nucleus, the ventral medial branch of the parabasal nucleus, and the central nucleus of the central nucleus is exacerbated in Parkinson’s patients (Banwinkler et al., 2022).Therefore, in addition to focusing on repairing the structure and function of the relevant brain areas after the onset of the disease, it is essential to prevent the extension of the lesion. Acceptance music therapy can be used as an adjunctive program to intervene in the early stages of the disease due to its therapeutic advantages of “simplicity of use and good compliance.”

### 3.2 The “PFC-Hip-Amy” circuit influences the development of psychiatric disorders

The “PFC-Hip-Amy” circuit, as a key circuit for emotional processing, plays an undisputed role in the treatment of psychiatric disorders. Disorders such as autism, schizophrenia, and post-traumatic stress disorder are associated with abnormal functioning of this circuit.

Studies have shown that damage to the “medial Prefrontal Cortex (mPFC)-Hip-Amy” synapse is the main reason for the loss of social interaction and intellectual developmental deficits in children with autism (Welsh et al., 2023). Asymmetry in hippocampal shape is significantly increased in children with autism and is accompanied by delayed growth and restricted development of hippocampal neurons (Richards et al., 2020). At the same time, the amygdala’s reduced responsiveness to stimuli and the persistent activation of sad-face habituation lead to the development of social deficits and even destructive and aggressive behaviors in children (Ibrahim et al., 2019). In contrast, Zeng et al. (2022) found that brain function in patients with schizophrenia demonstrated that both the amygdala and hippocampus showed hyperactivation, with reduced activity in the granular dorsolateral Prefrontal Cortex (dlPFC) and mPFC.

In addition, down-regulation of fear learning and threat detection circuits centered on the amygdala, and up-regulation of circuits regulating emotion and situational executive processing such as the PFC and the hippocampus can be effective in ameliorating PTSD, reducing the number of times that patients’ fearful memories are reproduced, and mitigating vigilance and avoidance responses (van Rooij et al., 2021).

Thus, different psychiatric disorders show different levels of activity in the “mPFC-Hip-Amy” brain region. This suggests that the right melodic style should be chosen for each psychiatric disorder. For example, soothing and soft music is used to reduce amygdala overactivation, which can be beneficial for people with hyperactive moods such as schizophrenia. On the other hand, relaxing music is suitable for application to patients with autism and post-traumatic stress disorder, which can boost their mental energy to cope with external challenges.

### 3.3 The “PFC-Hip-Amy” circuit influences the development of the co-morbidity of neuropsychiatric disorders

Mental illness is often accompanied by concomitant neurological disorders, a comorbidity that not only shortens patients’ life expectancy but also increases the risk of death (Momen et al., 2022). For example, patients with Alzheimer’s disease show early symptoms of major depression such as “inner loneliness, easy fatigue, indecisiveness.” This is rooted in the dysregulation of the “PFC-Hip-Amy” circuits by common genes (Ciart, Grin3b, Nr1d1, and Mc4r) between the two diseases. In addition, microglia activation and release of IL-6 and IL-1β induces the emergence of neuroinflammation in the brain, triggering hippocampal sclerosis and amygdala hypertrophy, which in turn leads to a vicious cycle of epileptic and depressive seizures (Wen et al., 2023). In addition, Parkinson’s patients are often associated with anxiety, and brain imaging shows reduced gray matter volume in the amygdala and anterior cingulate cortex (ACC), increased functional connectivity (FC) between the amygdala, hippocampus and mPFC, and reduced FC between the lateral Prefrontal Cortex (lPFC), Hip, and Amy (Carey et al., 2021). Sleep disorders are the most frequent problems in neurological patients, often inducing symptoms such as depression, anxiety and fatigue (Reeve et al., 2019). Muñoz-Torres et al. proposed that the “PFC-Hip-Amy” circuit exhibits different activity patterns during various sleep states such as wakefulness (W), Non-Rapid Eye Movement (NREM), and Rapid Eye Movement (REM) sleep. During NREM sleep, there is increased activity synchrony between the hippocampus and the prefrontal cortex, as well as between the amygdala and the temporal lobe. In contrast, during REM sleep, there is heightened activity synchrony between the amygdala and the frontal lobe, as well as between the hippocampus and the temporal lobe (Munoz-Torres et al., 2023).

**FIGURE 3:**
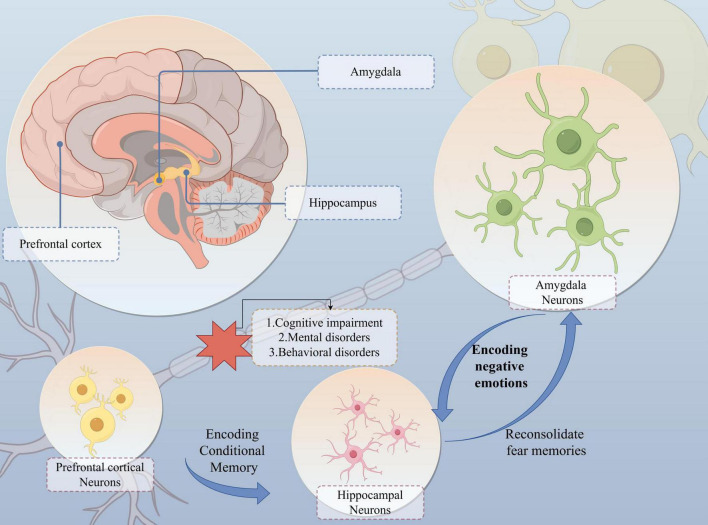
The “PFC-Hip-Amy” circuit influences the development of the NPDs. The prefrontal cortex, hippocampus and amygdala are interconnected and coordinate neural activity. In particular, the “amygdala-hippocampus” connection is the main neural structure for fear- and threat-related behaviours and learning in mammals. Whereas the hippocampus and prefrontal cortex are responsible for encoding the formation of conditioned memories and dominate the reproduction of memories after they have faded, affecting the patient’s functions such as remembering objects, recognising orientation, and planning motor routes. Impairment of the function of this circuit results in cognitive, mental and behavioural abnormalities.

In summary, the “PFC-Hip-Amy” circuit plays a complex and crucial role in the development of comorbidities associated with NPDs, involving various aspects such as changes in neurotransmitter and gene expression, neuroinflammation, inter-regional information exchange, pain processing, and biopsychosocial pain management. Understanding the specific molecular mechanisms and pathophysiological changes underlying these functions is an important direction for future research, which can aid in better comprehending and treating NPDs and their comorbidities (Figure 3).

## 4 Common circuits and molecular mechanisms of music therapy intervention in neuropsychiatric disorders

Although the mechanisms by which MT intervenes in NPDs are quite complex, a multitude of literature provides direct or indirect evidence supporting the association between MT and the “PFC-Hip-Amy” neural circuitry (Cheung et al., 2019). Modern research indicates that, on one hand, music can enhance the plasticity of brain neurons by conducting through the auditory cortex, directly repairing damaged areas such as PFC, amygdala, hippocampus, and other brain regions (Strait and Kraus, 2014). On the other hand, music may indirectly improve the functional state of brain circuits by regulating neurotransmitters, inflammatory factors, and gut microbiota (Chanda and Levitin, 2013). The following summarizes the achievements of more in-depth research on phenotypes and mechanisms, and boldly speculates that glial cells and mitochondria may be potential targets for the MT of NPDs, providing new ideas for future experimental research (Merheb et al., 2019).

### 4.1 Direct effects of music on the “PFC-Hip-Amy” circuitry

A wealth of research has established that music has the capacity to stimulate various brain regions. For instance, it has been shown to enhance executive functions in individuals with traumatic brain injury by augmenting neuroplasticity within the PFC (O’Sullivan et al., 2023). At the physiological level, people can feel intense pleasure when listening to a surprising melody that exceeds their expectations, which is related to music-induced interactions between the amygdala, the hippocampus, and the auditory cortex (GBD 2021 Causes of Death Collaborators, 2024). Furthermore, brain circuits have a reverse recognition function for music. It has been noted that damage to brain regions, including the vmPFC, disrupts human emotional understanding of music and liaison with society, recognizing dissonance in tones and embodying neural representations of negative emotions (Belfi, 2021; Bravo et al., 2020). At the pathological level, the efficacy of music in decelerating the rate of neurological deterioration is significantly correlated with enhanced functional connectivity between the PFC and somatomotor areas (Siponkoski et al., 2020). Music also demonstrates considerable potential in restoring hippocampal and amygdala functions. For example, extended exposure to Mozart K. 448 has been shown to modulate seizure EEG spectral power in the hippocampus and to amplify the antiepileptic effects of subtherapeutic drug doses (Paprad et al., 2021). Fu et al. (2023) demonstrated that passive receptive music therapy can effectively alleviate anxiety and depression-like behaviors in mice subjected to chronic unpredictable stress, attributed to the inhibition of oxidative stress in the PFC and hippocampus, thus preventing further neural dysfunction in this circuit. Additionally, the rhythmic magnetic field generated by soothing music modulates long-term potentiation (LTP) signaling at Schaffer-CA1 synapses in the rat hippocampus, with the enhancement of synaptic plasticity in rhythmic magnetic fields produced by a musical frequency of 3,500 Hz being the most pronounced (Jin et al., 2023).

In summary, the hippocampus activates the superior temporal gyrus during the processing of melodic sequences, which then influences the PFC and amygdala, subsequently regulating the balance of glutamate (Glu) and gamma-aminobutyric acid (GABA) and promoting dopamine secretion (Ong et al., 2019; Putkinen et al., 2021). This sequence of events ultimately serves to achieve analgesic effects, suppress fear, and alleviate adverse emotions such as anxiety and depression.

### 4.2 Indirect effects of music on the “PFC-Hip-Amy” circuitry

**FIGURE 4:**
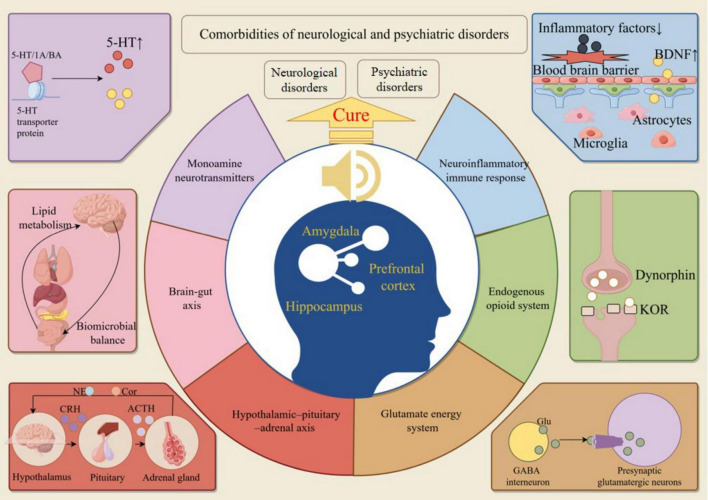
Music repairs the “PFC-Hip-Amy” circuit to cure NPDs. MT treats NPDs by regulating neurotransmitters, glutamatergic system, intestinal flora, inflammatory factors, endogenous opioid peptide system, and indirectly repairs the structure and function of the “PFC-Hip-Amy” circuit.

The “PFC-Hip-Amy” circuitry is engaged with monoamine dysregulation, the glutamatergic system, the gut-brain axis, and the neuroinflammatory immune response, all of which significantly impact the development and healing processes of NPDs (Sanacora et al., 2022). Scholars have attributed the therapeutic effectiveness of MT intervention in NPDs to these mechanisms, which are believed to facilitate the repair of brain region circuits and the improvement of related functions (Blood and Zatorre, 2001). The following content primarily explores the potential mechanisms by which music, through the modulation of the aforementioned mechanisms, repairs the “PFC-Hip-Amy” circuitry to improve the functional status of patients with NPDs (Figure 4).

#### 4.2.1 Monoamine neurotransmitters

Monoamine neurotransmitters mainly include 5-hydroxytryptamine (5-HT), norepinephrine (NE) and dopamine (DA). Abnormal secretion of monoamine neurotransmitters is an important pathological mechanism in NPD and severely affects the function of the PFC, hippocampus and amygdala and synaptic signaling in all three networks (Cheng and Bahar, 2019; Cools and Arnsten, 2022). Specifically, spatial learning and memory enhancements were associated with increased concentrations of monoamine neurotransmitters in the hippocampus and PFC (Piechal et al., 2023). 5-HT transporter protein binds to receptors such as 5-HT1A/1B, which can increase 5-HT levels. It also repairs brain regions such as the PFC, hippocampus and amygdala by releasing the inhibitory state of GABA interneurons, activating GABA pyramidal neurons and stimulating the rapid release of BDNF in the brain (Li, 2020).

Many studies have provided strong evidence that MT regulates monoamine neurotransmitter secretion. For example, MT combining improvisation with passive listening to music was effective in elevating serum 5-HT levels in children with Attention Deficit Hyperactivity Disorder (ADHD) and adolescents with depression, and enhanced their ability to cope with stress (Park et al., 2023). Moreover, music can directly induce the release of DA, oxytocin and 5-HT in the brain and promote the recovery of attention, imagination, creativity and emotional memory in patients with brain injury (Speranza et al., 2022). In addition, the autonomic nervous system (ANS) and the hypothalamic-pituitary-adrenal (HPA) axis are also modulated by music (Sittler et al., 2021). A study based on vibroacoustics noted that monochordal musical stimulation induced an increase in salivary cortisol levels in patients with psychosomatic disorders, which in turn led to a sustained state of relaxation (Sandler et al., 2017). Elevated NE in the auditory cortex enhances sensory memory, and elevated DA levels can enhance the incentive salience of communication signals (Barr et al., 2021; Chen and Sakata, 2021).

Therefore, although the above studies seem to be showing that music therapy can restore monoamine neurotransmitters in the “PFC-Hip-Amy” circuit to normal metabolic levels, the specific effect of music on the level of monoamine neurotransmitters is still controversial. For example, a study of musical interventions on prenatal fear states in chicks showed that 65–75 dB of music and noise stimuli had no significant effect on cortisol (CORT) and 5-serotonin (5-HT) concentrations in chicks (*p* > 0.05). However, music and noise stimulation of 85–95 decibels significantly reduced CORT concentration (*p* < 0.05), but had no significant effect on 5-HT concentration (*p* > 0.05) (Zhao et al., 2022). Another study found that auditory stimulation significantly increased dopamine (DA) and 5-hydroxyindoleacetic acid (5-HIAA) concentrations in adult male Wistar rats, but had no effect on dihydroxyphenylacetic acid (DOPAC) or 5-HT levels (Moraes et al., 2018). These findings suggest that there may be differences in the effects of music on humans and animals, which may be related to human cognition and cultural identity of musical emotions (Fram, 2023). From the perspective of physiological structure, the cerebral cortex development of chicks and rats is quite different from that of humans, so more suitable animal models should be selected for future research (Penna et al., 2021). In addition, these phenomena also suggest that the mechanism of music’s influence on brain function and structure may be different from that of drug therapy, providing a new direction for future research.

#### 4.2.2 The glutamatergic system

The glutaminergic system plays a crucial role in the PFC, hippocampus and amygdala, which play a central role in regulating stress-related emotions of sleep, anxiety and fear. Many scholars believe that the balance of glutaminergic system is an important marker of regulating brain function in NPDs. In the field of MT, attention has also been paid to the regulatory significance of this system. Studies have shown that activation of the glutaminergic system mediates neural adaptations in the PFC, hippocampus, and amygdala that contribute to the emergence of addiction symptoms (Volkow et al., 2019).

However, training about music rhythm perception and improvisation ability can inhibit the excitability of GABAergic neurons, enhance the Glu/GABA ratio, and promote the blood flow metabolism in the above three brain regions (Honda et al., 2023; Zhang et al., 2022). Direct evidence suggests that music promotes Glu secretion in the hippocampus and induces induction of glutaminergic pathways, followed by the production of GRIK5, DLG4, and NMDA 1 protein which in turn activate dopaminergic neurons, and ultimately promotes the production of 5-HT, which dramatically reduces somatic pain sensation in depressed mice (Mao et al., 2022). In addition, music may activate vmPFC ventral projections to the amygdala and reduce the concentration of Glu unidirectional inputs from the dmPFC to basolateral amygdala neurons as a means of sustained alleviation of anxiety-like behaviors in chronic restraint stress mice (Liu et al., 2020; Yan and Rein, 2022).

Although research on the correlation between music and the glutamatergic system is relatively scarce, it is noteworthy that studies have shown that under the context of Chinese Five Elements music, different melodies of music may have varying impacts on the levels of amino acid neurotransmitters within organisms (Hao et al., 2020). This finding suggests that a deeper investigation into the types and patterns of music, along with the establishment of standards, could be crucial for music therapy to provide targeted assistance in treating NPDs accompanied by various emotional disorders such as fear, anxiety, depression, mania, and sadness (McCoyd, 2022). By understanding how music influences the glutamatergic system, we can better utilize music therapy to regulate emotional and cognitive functions, thereby offering more personalized and effective treatment plans for patients with NPDs.

#### 4.2.3 Brain-gut axis

As a bidirectional signaling pathway between the gastrointestinal tract and the central nervous system, the brain-gut-microbiota axis interacts in the pathophysiology of NPD (Generoso et al., 2021). Deficiencies in the gut microbiota reduce lipid metabolism in relevant brain regions, leading to brain circuit dysfunction and biobehavioural disorders (Liu G. et al., 2022). Conversely, neural development and transmission depend on gut microbial shaping, and elevated plasma intestinal fatty acid-binding proteins and abnormal lipid metabolism predict the onset of some NPDs (Socala et al., 2021). Research has confirmed that the balance of the gut microbiota influences the synaptic activity of neurons in the mPFC and is also involved in the development of the amygdala and hippocampus (Trzeciak and Herbet, 2021; Wang et al., 2022). Consequently, the brain-gut axis may play a significant role in the “PFC-Hip-Amy” circuitry.

The regulatory effect of music on the gut microbiota has been confirmed by a vast body of literature. In a randomized controlled trial, it was shown that MT restored the disturbed state of gut flora in cancer patients, further improving anxiety symptoms (Sun et al., 2023). Similar findings have been corroborated through animal studies. Research indicates that musical stimulation during feeding can significantly enhance the levels of beneficial bacteria, such as Firmicutes and Lactobacillus, in mice. Concurrently, this intervention has been shown to reduce the prevalence of pathogenic bacteria, thereby restoring a balanced and homeostatic state within the murine gut microbiota (Niu et al., 2023). Another study has reached more specific conclusions. After exposing two groups of mice to music and white noise, respectively, it was observed that music may have a positive impact on the gut microbiota, antioxidant activity, and immune function in mice, whereas white noise may exert detrimental effects (Zhang Z. et al., 2023). This finding suggests that not all types of music have a positive effect on mice, which inspires us to further investigate the different sound waves and energies conveyed by various types of music. Such detailed research could help us gain a deeper understanding of the mechanisms by which music affects NPDs.

In summary, current research has predominantly focused on the impact of music on gut microbiota, with relatively fewer studies exploring the role of gut microbiota as a potential mechanism in the comorbidities of neurological and psychiatric disorders (Hasic Telalovic and Music, 2020). Therefore, future research directions could concentrate on the correlation between NPDs and the gut-brain axis, as well as in-depth studies on how music affects mechanisms related to the gut-brain axis. Such research would provide more robust scientific evidence for the use of music intervention in NPDs, thereby deepening our understanding and application of the potential of music therapy.

#### 4.2.4 Neuroinflammatory immune response

The interaction between endogenous retroviral activation and inflammation is a prevalent pathogenic mechanism in both neurological and psychiatric disorders. Immune processes are crucial for maintaining central nervous system (CNS) stability, self-repair, and cognitive reserve. NPDs are often triggered by microglial activation, the production of pro-inflammatory cytokines, and disruptions to the blood-brain barrier (Gruchot et al., 2023; Pape et al., 2019). In particular, autophagy in microglia is stimulated by blocking α-synuclein and NLRP3 inflammatory vesicles, which helps to preserve brain homeostasis (Lv et al., 2023). Numerous therapeutic strategies for NPD focus on diminishing neuroinflammatory responses within the hippocampus and amygdala-derived microglia (Butler et al., 2020). For instance, enhancing microglial function can suppress the release of pro-inflammatory cytokines such as TNF-α, IL-6, and IL-1β in the PFC and hippocampus, thereby alleviating stress-induced depressive-like behaviors (Duan et al., 2022).

MT plays an excellent role in mitigating the neuroinflammatory immune response. Research indicates that MT can help lower the elevated levels of inflammatory factors triggered by acute stress (Johnson et al., 2018). This effect may be mediated by activating microglia, reducing the severity of lesions in the PFC, and enhancing BDNF expression in the hippocampus (Moschonas et al., 2023). Community mental health activities often involve group-based music therapy, a group interaction that is more beneficial to physical and mental health. For example, group drumming reduces people’s IL4, IL6, IL17, TNFα, and cortisol levels and improves the wellbeing of people in the community (Fancourt et al., 2016). Furthermore, implementing music therapy through singing in patients with postpartum depression has demonstrated remarkable efficacy. It not only alleviates depressive symptoms but also leads to a reduction in inflammatory factor levels (Estevao et al., 2021). This approach highlights the multifaceted benefits of music therapy in addressing both psychological and physiological aspects of health.

Although music has positive effects on human health, improper use can transform it into noise, potentially causing harm. A study has demonstrated that noise can induce the production of autoantibodies, such as anti-Hsp70 and anti-Hsp60, leading to the occurrence of noise-induced hearing loss (NIHL), and can also have adverse effects associated with other immune-related diseases, such as certain autoimmune diseases and non-Hodgkin lymphoma (Zhang et al., 2020). The latest research indicates that 14 consecutive days of musical stimulation significantly reduced the relative expression levels of NF-κB protein in broiler chicks following acute noise stress (*P* < 0.05) (Wang et al., 2024). It is suggested that music may mitigate the inflammatory response in immune organs caused by acute noise stress through the NF-κB signaling pathway.

The aforementioned studies have revealed the dual effects of sound energy, where noise may impose detrimental impacts on living organisms, while soothing music may mitigate adverse effects. The research also discovered that MT can regulate inflammatory responses, providing new avenues for future research. This finding may aid in applying music therapy to the treatment of acute inflammatory diseases, including colds and flu, as well as chronic conditions such as pain, diabetes, and cancer, contributing to human health and wellbeing.

#### 4.2.5 Endogenous opioid peptide systems

Endogenous opioid peptides are concentrated in the “PFC-Hip-Amy” loop, and are directly or indirectly involved in regulating glial cell development and nerve fiber myelin production (Velasco et al., 2021). The release of naturally occurring opioid-like active substances into the circulatory system in mammals is capable of activating opioid receptors involved in many emotional and physiological responses (Wang Y. et al., 2023). Release of dynorphins in the mPFC and disrupt cognitive function by activating local kappa opioid receptors (KOR) (Abraham et al., 2021). The endogenous opioid peptide system is also implicated in the neuroinflammatory response. The binding of endogenous opioid peptides to μ-opioid receptors (MOR) promotes neuroinflammation and microglial activation in the limbic system, including the “PFC-Hip-Amy,” inducing inflammatory pain (Cuitavi et al., 2023). Furthermore, up-regulation of the dynorphin/KOR system in the amygdala contributes to the emergence of depression following the stress of chronic social failure. This reveals that KOR antagonists may serve as potential substances for the prevention and treatment of chronic post-stress depression (Zan et al., 2022).

The modulation of endogenous opioid peptide secretion by music has become an accepted proposition (Fritz et al., 2017). Sun et al. (2022) found a significant correlation between MOR availability and blood oxygen-dependent level signaling in the amygdala and auditory cortex. Endogenous MOR availability is reduced in the amygdala of depressed patients (Nummenmaa et al., 2020). Music-induced pleasure is precisely positively correlated with the activity response of the amygdala, which regulates the release of endogenous opioid peptides, such as endorphins (END) in the amygdala, thereby ameliorating bad mood (Cheung et al., 2019). For example, MT in the form of singing training can upregulate oxytocin and endorphins, which improves immune function and increases a singer’s sense of wellbeing (Kang et al., 2018). Another study found that music can reduce pain responses in preterm infants by increasing beta-endorphin concentrations (Qiu et al., 2017).

All of the above studies suggest that MT has the potential to improve many of the dysfunctions of NPD by improving the levels of endogenous opioid peptides in the PFC, hippocampus, and amygdala, or by blocking binding to opioid-like receptors, which affects the function of the “PFC Hip-Amy” circuit.

In summary, MT has demonstrated promising effects in the treatment of NPDs, with its mechanisms potentially being closely linked to monoamine neurotransmitters, the glutamatergic system, the brain-gut axis, inflammatory responses, and the endogenous opioid system. However, it remains unclear whether the mechanisms of MT are consistent with those of commonly used cognitive-behavioral therapies and pharmacological treatments. If the mechanisms are indeed aligned, there is a possibility that MT could serve as an alternative intervention to medication. Conversely, if the mechanisms differ, there is a risk that MT could conflict with medications, potentially leading to suboptimal outcomes. These are important considerations that warrant further research and exploration.

### 4.3 “PFC Hip-Amy” circuit as potential targets based for music intervent neuropsychiatric disorders

A substantial amount of research indicates that music can exert a beneficial influence on the “PFC Hip-Amy” circuit. However, there has been comparatively little focus on the specific targets of music therapy within this circuit. The following discussion aims to investigate neuroglia, mitochondria, and microRNA gene sequences as potential targets for music therapy interventions in NPD.

#### 4.3.1 Neuroglia

Neuroglia plays an important role in both synaptic transmission in the brain and signaling in the auditory pathway (Zhou et al., 2019). As an important target of NPD, neuroglia is involved in the whole process of brain function from injury to repair, including neuroinflammation, blood-brain barrier integrity, glutamate toxicity, blood oxygen metabolism, and nerve regeneration (Kono et al., 2021). Interestingly, glial cells are also involved in processing complex sound information received by humans including speech and music. Studies have shown that hair cells and neuronal axons in the upstream auditory pathway interact with glial cells and accurately capture and process valid information in sub-millisecond increments to detect the timing, frequency and intensity of sound signals (Kohrman et al., 2021). Moreover, in vibroacoustics, different styles of music can produce different rhythmic impulse stimuli, and pleasant music plays a key role in activating protein kinases in glial cells (Mozaffari et al., 2023). Studies have shown that musical pulse stimulation facilitates the promotion of significant expression of antioxidants such as glutathione peroxidase-1 (GPX-1), catalase (CAT), and superoxide dismutase 1 (SOD1), and reduces oxidative stress in neuronal cells (Bartel and Mosabbir, 2021). It is well known that beautiful music promotes dopamine secretion. It has been pointed out that fast Ca^2+^ signaling in astrocytes in the PFC is dopaminergically regulated and promotes functional recovery of the PFC by triggering α1-adrenergic receptors (Pittolo et al., 2022). Meanwhile, music may attenuate neuronal apoptosis by inhibiting TRPV4 expression in hippocampal glial cells, which reduces TRPV4-mediated Ca^2+^ inward flow and NF-κB nuclear translocation, and decreases IL-1β and TNF-α levels (Yang X. L. et al., 2019). Therefore, glial cells may play a central role as an important target in the repair of the “PFC Hip-Amy” circuit in MT and in the treatment of NPD.

In addition, noise induces enhanced release of ATP and glutamate by astrocyte Cx43 HC, leading to learning and memory deficits in patients (Zhang Z. et al., 2023). This phenomenon sheds light on the fact that glial cells may play a bidirectional regulatory role similar to that of a crossroads. Therefore, during music therapy, the therapist should strictly control the melody, style, and playing volume of the selected tracks. If the patient is unable to accept the track, the pleasant sound will become noise, which in turn will aggravate the patient’s neurological impairment. On the other hand, do sound vibration frequencies resonate directly with glial cells via mechanical waves? Do different frequencies affect the expression of different signals in glial cells? The above queries require further exploration and research.

#### 4.3.2 Mitochondria

Mitochondria are considered to be the gateway for receiving, processing and integrating external energy and information to maintain cellular homeostasis (Sulkshane et al., 2020). Feng et al. (2022a) found that the mitochondrial function and antioxidant capacity of mammalian cells are altered by different energetic stimuli such as sound waves over a relatively short period of time. Moreover, traditional Chinese five-element music enables mitochondria to produce adenosine triphosphate (ATP) and glutathione (GSH) as a means of accelerating cell growth rates and inhibiting oxidative stress. In contrast, mitochondrial oxidation of cells under the influence of heavy metal music was significantly increased and cell viability was famously reduced (Feng et al., 2022b). This study also provides mechanistic evidence to support the previously mentioned idea that different music has opposite effects.

At the same time, mitochondria also play an important role in intracellular signaling in neural connections to skeletal muscle (Arosio et al., 2023). NPD has somato-motor and behavioral problems in addition to mental and cognitive dysfunction. It has been found that mitochondrial transplantation promotes the survival of neurons and astrocytes and elevates BDNF to protect and repair the damaged brain (Zhao et al., 2021). In another study, it was noted that music was able to activate the PFC FNDC5/BDNF pathway, thereby increasing mitochondrial autophagy and remodeling, which ultimately had a promising effect on improving social inhibition in mice with Rett syndrome (Hung et al., 2021). Therefore, it is hypothesized that the microscopic mechanism of musical intervention in NPD is related to an increase in the number and quality of healthy mitochondria and acts on glial cells to repair the “PFC Hip-Amy” circuit.

#### 4.3.3 MicroRNA

MicroRNAs (miRNAs) are a class of non-coding RNAs that are important regulators of neuronal growth and repair, and play a key role in both the structure and function of nerves (Martins and Schratt, 2021). Abnormal miRNA expression, as well as miRNA-mediated gene regulation, have been closely linked to both neurological dysfunction and psychiatric disorders (Sanacora et al., 2022). Studies have shown that microRNAs are expressed or inhibited in the PFC, hippocampal neurons and amygdala (de Sena Cortabitarte et al., 2018). MicroRNAs are also involved in the regulation of mitochondrial autophagy, monoamine oxidase activity, and stress response. In conjunction with μ-opioid receptors, dopamine signaling pathways, oxytocin and taurine levels act on the amygdala to regulate neural development and repair (Seo and Anderson, 2019). In addition, microRNAs may be key targets on the brain-gut axis. The study shows that this gene acts as an important communication channel between the gut microbiome and the host, regulating the integrity of the intestinal epithelium and the blood-brain barrier. Meanwhile, hippocampal microRNA-26a-3p deletion is participating in the amelioration of neuroinflammation and behavioral deficits in rats through the p38 MAPK signaling pathway (Datta et al., 2023). Thus, this gene is involved in many physiopathological mechanisms of NPDs and may be a major target for many therapeutic options to intervene in NPDs.

Listening to music has been shown to upregulate microRNA-132, increase hippocampal neuronal plasticity, promote CNS myelin formation, and further regulate striatal dopamine levels (Nair et al., 2021). This may be the main potential mechanism by which music can improve memory capacity and cognitive function. A similar conclusion was reached in a study with musicians, in which long-term musical performance favored the up-regulation of microRNAs (miR-30d, miR-92a, and miR-222), apoptosis of hippocampal neurons was inhibited, and auditory ability and neurological function were both greatly enhanced (Nair et al., 2019). Finally, the music-promoted up-regulation of microRNA-132 expression may be related to the activation of the reward pathway, which may be the underlying reason for the increase in transmitters such as 5-HT, DA, and endorphins in NPD patients after listening to music (Jarvela, 2018). Thus, music therapy can be ameliorative in many of the psychosomatic and somato-motor dysfunctions produced by NPDs. The underlying cause of the phenomenon is that music regulates microRNA expression.

## 5 Summary and outlook

Music, a borderless language accessible to all, has shown remarkable advantages in alleviating the mental, motor, and sensory disturbances of neurological and psychiatric disorders, enabling individuals of diverse genders and ages to reclaim their social roles. Based on the above discussion, we consider that: (1) MT has a positive effect on improving cognition, mood, and motor functions in patients with NPDs. (2) The “PFC-Hip-Amy” circuit is a common key circuit involved in the pathogenesis of various NPDs. (3) The intervention of music in various NPDs may achieve its effects by modulating the function of the “PFC-Hip-Amy” circuit; (4) MT may restore the “PFC-Hip-Amy” circuit and alleviate symptoms of neuropsychiatric disorders by regulating the secretion of monoamine neurotransmitters, the glutamatergic system, the brain-gut axis, neuroinflammatory immune responses, and the endogenous opioid system, thereby achieving a balance in their functions; (5) Glial cells, mitochondria, and microRNA gene sequences may be key targets for MT in repairing the “PFC-Hip-Amy” circuit.

**FIGURE 5:**
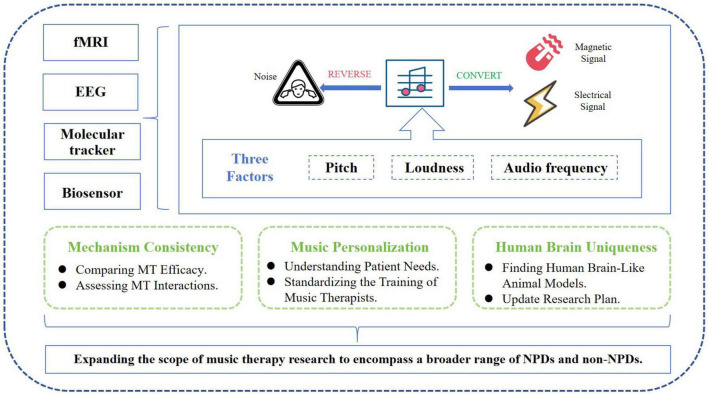
Prospects of music therapy research. In the future, research will concentrate on the three core elements of music (pitch, volume, and tone) to investigate the therapeutic effects and mechanisms of MT across a spectrum of diseases, encompassing both neurological and non-neurological conditions. Employing sophisticated technologies and suitable animal models, we will utilize fMRI, EEG, molecular tracers, and biosensors to delve deeper into the study of MT’s impact on NPDs and non-NPDs.

National and international research has emphasized the replication and broad applicability of music therapy, focusing on the direct relevance of music’s auditory neural pathways. However, there remain several questions that await our resolution. For example, there is a relative dearth of studies examining the direct effects of the rhythmic magnetic fields generated by music on specific neural circuits or neurotransmitters. Does music exert its influence by altering wave frequencies and magnetic fields? Deciphering this complex mechanism not only addresses the challenges faced by hearing-impaired individuals in accessing MT but also opens up new avenues for in-depth and expanded research in MT (Figure 5). Moreover, the sequential activation and regulation of neurons in different brain regions by music have not been fully elucidated. Resolving this issue would be beneficial for determining the duration of clinical MT interventions and quantifying them to precisely control targeted brain areas and improve specific neuropsychiatric functional impairments. Ultimately, the optimization of MT outcomes is contingent upon the standardized practices and seasoned skills of music therapists. Consequently, a deeper understanding of the specific mechanisms behind MT can serve as a guide for therapists to more scientifically engage with various elements such as “volume, pitch, frequency, timing, and music selection,” thereby enhancing the therapeutic efficacy.

In the future, we plan to utilize advanced tools such as functional magnetic resonance imaging (fMRI), molecular tracing, or biosensors to analyze the direct or indirect relationships between music and the “PFC-Hip-Amy” circuit. This will allow us to reveal causal links in a targeted way and further investigate the underlying mechanisms of MT.
